# Integrative Brain Transcriptome Analysis Reveals Region-Specific and Broad Molecular Changes in *Shank3*-Overexpressing Mice

**DOI:** 10.3389/fnmol.2018.00250

**Published:** 2018-08-31

**Authors:** Chunmei Jin, Hyojin Kang, Jae Ryun Ryu, Shinhyun Kim, Yinhua Zhang, Yeunkum Lee, Yoonhee Kim, Kihoon Han

**Affiliations:** ^1^Department of Neuroscience, College of Medicine, Korea University, Seoul, South Korea; ^2^Department of Biomedical Sciences, College of Medicine, Korea University, Seoul, South Korea; ^3^Supercomputing Center, Korea Institute of Science and Technology Information, Daejeon, South Korea; ^4^Department of Anatomy, College of Medicine, Korea University, Seoul, South Korea

**Keywords:** Shank3, mPFC, striatum, transcriptome, myelin, GPR85, ribosome

## Abstract

Variants of the SH3 and multiple ankyrin repeat domain 3 (*SHANK3*) gene, encoding excitatory postsynaptic core scaffolding proteins, are causally associated with numerous neurodevelopmental and neuropsychiatric disorders, including autism spectrum disorder (ASD), bipolar disorder, intellectual disability, and schizophrenia (SCZ). Although detailed synaptic changes of various *Shank3* mutant mice have been well characterized, broader downstream molecular changes, including direct and indirect changes, remain largely unknown. To address this issue, we performed a transcriptome analysis of the medial prefrontal cortex (mPFC) of adult *Shank3*-overexpressing transgenic (TG) mice, using an RNA-sequencing approach. We also re-analyzed previously reported RNA-sequencing results of the striatum of adult *Shank3* TG mice and of the prefrontal cortex of juvenile *Shank3^+/^*^Δ^*^C^* mice with a 50–70% reduction of Shank3 proteins. We found that several myelin-related genes were significantly downregulated specifically in the mPFC, but not in the striatum or hippocampus, of adult *Shank3* TG mice by comparing the differentially expressed genes (DEGs) of the analyses side by side. Moreover, we also found nine common DEGs between the mPFC and striatum of *Shank3* TG mice, among which we further characterized ASD- and SCZ-associated G protein-coupled receptor 85 (*Gpr85*), encoding an orphan *Gpr* interacting with PSD-95. Unlike the mPFC-specific decrease of myelin-related genes, we found that the mRNA levels of *Gpr85* increased in multiple brain regions of adult *Shank3* TG mice, whereas the mRNA levels of its family members, *Gpr27* and *Gpr173*, decreased in the cortex and striatum. Intriguingly, in cultured neurons, the mRNA levels of *Gpr27*, *Gpr85*, and *Gpr173* were modulated by the neuronal activity. Furthermore, exogenously expressed GPR85 was co-localized with PSD-95 and Shank3 in cultured neurons and negatively regulated the number of excitatory synapses, suggesting its potential role in homeostatic regulation of excitatory synapses in *Shank3* TG neurons. Finally, we performed a gene set enrichment analysis of the RNA-sequencing results, which suggested that Shank3 could affect the directional expression pattern of numerous ribosome-related genes in a dosage-dependent manner. To sum up, these results reveal previously unidentified brain region-specific and broad molecular changes in *Shank3*-overexpressing mice, further elucidating the complexity of the molecular pathophysiology of *SHANK3*-associated brain disorders.

## Introduction

SH3 and multiple ankyrin repeat domain 3 (*SHANK3*), also known as proline-rich synapse-associated protein 2 (*ProSAP2*), is a gene that encodes excitatory synaptic core scaffolding proteins that organize the macromolecular protein complex of the postsynaptic density (PSD) (Naisbitt et al., 1999; Sheng and Kim, 2000). Clinically, deletions of the chromosomal region containing *SHANK3* cause Phelan–McDermid syndrome (Wilson et al., 2003; Costales and Kolevzon, 2015; Harony-Nicolas et al., 2015), and a variety of point mutations and small deletions of *SHANK3* have been causally associated with numerous neurodevelopmental and neuropsychiatric disorders, including autism spectrum disorder (ASD), intellectual disability, and schizophrenia (SCZ) (Grabrucker et al., 2011; Duffney et al., 2015), which have been modeled by several lines of knock-out and knock-in mouse models (Jiang and Ehlers, 2013; Yoo et al., 2014; Schmeisser, 2015; Monteiro and Feng, 2017). Moreover, duplications of *SHANK3* have also been found in patients with Asperger’s syndrome, attention-deficit hyperactivity disorder (ADHD) (Moessner et al., 2007), bipolar disorder (Han et al., 2013), and SCZ (Failla et al., 2007), and the transgenic mice that mildly overexpress Shank3 proteins (∼50%) display manic-like hyperkinetic behaviors and spontaneous seizures (Han et al., 2013; Choi and Han, 2015; Lee et al., 2018). These results indicate that proper expression and function of Shank3 are critical for normal synaptic development and function. Indeed, Shank3-dependent molecular, structural, and functional changes of excitatory synapses have been deeply characterized from *in vitro* and *in vivo* studies to elucidate some of the key underlying pathophysiological mechanisms (Han et al., 2013; Wang et al., 2016) and to provide potential therapeutic approaches for *SHANK3*-associated brain disorders, mainly ASDs (Bozdagi et al., 2013; Shcheglovitov et al., 2013; Duffney et al., 2015; Bidinosti et al., 2016; Vicidomini et al., 2016; Wang et al., 2016).

In contrast, it still remains largely unknown how different variants of a single gene, *SHANK3*, can lead to diverse phenotypic outcomes or clinical symptoms. Possible explanations could be that *SHANK3* expresses numerous Shank3 protein isoforms due to alternative splicing and multiple internal promoters, and that different brain regions express different combinations and levels of these Shank3 isoforms (Wang et al., 2014). Furthermore, we recently demonstrated that Shank3 protein interactomes of different brain regions consist of both brain region-specific (major portion) and common interactors (minor portion), which, together with the isoform diversity, suggest that alterations of Shank3 expression or function could have some distinct effects in different brain regions (Lee et al., 2017b). Supporting this hypothesis, it was reported that the functional changes of different brain regions could vary even in a single *Shank3* mutant mouse line (Peca et al., 2011; Lee et al., 2015; Zhou et al., 2016). Nevertheless, these results are so far limited to the characterizations of synaptic changes in *Shank3* knock-out mice. Broader downstream molecular changes, including direct and indirect changes, of Shank3 that might be possible even in non-neuronal cell types remain scarcely investigated.

To address this issue, in this study, we performed a transcriptome analysis of the medial prefrontal cortex (mPFC) of *Shank3*-overexpressing transgenic (TG) mice (Han et al., 2013) and compared the result with previously reported transcriptome analyses of the striatum of *Shank3* TG mice (Lee et al., 2017d) and the prefrontal cortex (PFC) of *Shank3* heterozygous (*Shank3^+/^*^Δ^*^C^*, heterozygous mice for C-terminal exon 21 deletion of *Shank3*) mice (Duffney et al., 2015; Qin et al., 2018). We focused on the mPFC and striatum for several reasons. The mPFC has well-established roles in top-down regulatory control over various subcortical nuclei involved in regulating emotional, social, and cognitive behaviors (Russo and Nestler, 2013; Riga et al., 2014), which are impaired in brain disorders associated with *SHANK3* mutations. The striatum is a key component of the brain motor and reward systems (Balleine et al., 2007; Russo and Nestler, 2013), abnormalities of which may contribute to behavioral symptoms observed in ASD, bipolar disorder, or SCZ. Furthermore, Shank3 is highly expressed in the mPFC and striatum (Monteiro and Feng, 2017), and indeed, molecular, cellular, and electrophysiological defects in the mPFC and striatal neurons are observed in numerous rodent models having *Shank3* mutations (Peca et al., 2011; Lee et al., 2015; Harony-Nicolas et al., 2017; Bey et al., 2018).

From integrative analyses and experimental validations, we found that the expression levels of myelin-related genes were downregulated specifically in the mPFC, but not in the striatum or hippocampus, of *Shank3* TG mice. Meanwhile, the expression of *Gpr85*, encoding an orphan G protein-coupled receptor, was increased in multiple brain regions of *Shank3* TG mice. We then further characterized the functional effects of this gene on excitatory synapses. Finally, we found that Shank3 affected the directional expression pattern of numerous ribosome-related genes in a dosage-dependent manner. To sum up, these results provide new insights into the complexity and heterogeneity of the molecular pathophysiology of *SHANK3*-associated brain disorders.

## Materials and Methods

### Mice

The enhanced green fluorescent protein (EGFP)*-Shank3* transgenic (TG) mice used in this study have been described previously (Han et al., 2013; Lee et al., 2017a,b,d). The male wild-type (WT) and TG mice were bred and maintained in a C57BL/6J background according to the Korea University College of Medicine Research Requirements, and all the experimental procedures were approved by the Committees on Animal Research at the Korea University College of Medicine (KOREA-2016-0096). The mice were fed and had access to water *ad libitum* and were housed under a 12-h light–dark cycle. For all experiments, “control WT mice” means WT littermates of the TG mice used for experiments.

### RNA Sequencing and Analysis

The mice (10- to 12-week-old male WT and *Shank3* TG) were deeply anesthetized with isoflurane and decapitated. The mPFC was dissected from each brain using a brain matrix (Alto, SA-2175, coronal 1 mm). Specifically, we put brains in contact with the front side (olfactory bulb side) of the matrix and cut off the first 2 mm of brains including the olfactory bulb. Then, we dissected out the next 2 mm of the remaining brain tissue. From the resulting coronal sections, we further dissected the mPFC area as shown in Figure 1A of Lee et al. (2017b). After dissection, the mPFC was immediately placed in a RNAlater solution (Ambion) and stored at 4°C overnight. The mPFC from two mice of same genotype was pooled to make one RNA sample, and a total three pairs of RNA samples (three WT and three *Shank3* TG; thus total six mice per each genotype) were processed for RNA sequencing. RNA extraction, library preparation, cluster generation, and sequencing were performed by Macrogen Inc. (Seoul, Korea). RNA samples for sequencing were prepared using a TruSeq Stranded mRNA LT Sample Prep Kit (Illumina) according to the manufacturer’s instructions. An Illumina’s HiSeq 2000 was used for sequencing to generate 101-bp paired-end reads (**Supplementary Table S1**). Raw data were submitted to the GEO (Gene Expression Omnibus) repository under the accession number GSE113368.

Transcript abundance was estimated with Salmon (v0.9.1) (Patro et al., 2017) in quasi-mapping-based mode onto the *Mus musculus* genome (GRCm38) with GC bias correction (–gcBias). Quantified gene-level abundance data were imported to R (v.3.6.0) with the tximport (Soneson et al., 2015) package, and differential gene expression analysis was carried out using R/Bioconductor DEseq2 (v1.19.11) (Love et al., 2014). Normalized read counts were computed by dividing the raw read counts by size factors and fitted to a negative binomial distribution. The *P*-values were first corrected by applying an empirical estimation of the null distribution using the R fdrtool (v.1.2.15) package and then adjusted for multiple testing with the Benjamini–Hochberg correction. Genes with an adjusted *P*-value of <0.05 were considered as differentially expressed. Volcano plots were generated using the R ggplot2 (v.2.2.1) package.

The gene ontology (GO) and Kyoto Encyclopedia of Genes and Genomes (KEGG) pathway analyses were performed using DAVID software (version 6.8) (Huang da et al., 2009). Mouse gene names were converted to human homologs using the Mouse Genome Informatics (MGI) database^1^.

To define “myelin-related genes,” a total of 333 human and mouse genes were extracted from five query terms (“central nervous system myelination,” “myelination,” “structural constituent of myelin sheath,” “myelin sheath,” and “myelin sheath adaxonal region”) from AmiGO^2^.

Gene set enrichment analysis (GSEA)^3^ (Subramanian et al., 2005) was used to determine whether *a priori*-defined gene sets would show statistically significant differences in expression between *Shank3* TG and WT mice. Enrichment analysis was performed using GSEAPreranked (gsea-3.0.jar) module on gene set collections H (Hallmark gene sets; 50 gene sets) and CP (KEGG; 186 gene sets) downloaded from Molecular Signature Database (MSigDB) v6.1^4^. GSEAPreranked was applied using the list of all genes expressed, ranked by the fold change, and multiplied by the inverse of the *P*-value with recommended default settings (1,000 permutations and a classic scoring scheme). The false discovery rate (FDR) was estimated to control the false-positive finding of a given normalized enrichment score (NES) by comparing the tails of the observed and null distributions derived from 1,000 gene set permutations. The gene sets with an FDR of <0.05 were considered as significantly enriched.

### RNA Purification and qRT-PCR

Real-time quantitative reverse transcription PCR (qRT-PCR) was performed as described previously (Kim et al., 2016; Lee et al., 2017a). In brief, total RNA was extracted from the brain regions of WT and *Shank3* TG mice or cultured rat neurons using an miRNeasy Mini Kit (Qiagen) according to the manufacturer’s instructions. Two micrograms of total RNA were used for the cDNA synthesis using iScript^TM^ cDNA Synthesis Kit (Bio-Rad). Target mRNAs were detected and quantified by a real-time PCR instrument (CFX96 Touch, Bio-Rad) using SYBR Green master mix (Bio-Rad). The results were analyzed using the comparative *C*t method normalized against the housekeeping gene *Gapdh*. The primer sequences for real-time PCR are as follows:

Mouse *Plp1* forward 5′ CCCACCCCTATCCGCTAGTT 3′,

reverse 5′ CAGGAAAAAAAGCACCATTGTG 3′

Mouse *Myrf* forward 5′ TGGCAACTTCACCTACCACA 3′,

reverse 5′ GTGGAACCTCTGCAAAAAGC 3′

Mouse *Mobp* forward 5′ AACTCCAAGCGTGAGATCGT 3′,

reverse 5′ CTCGGTCACTTCTTCCTTGG 3′

Mouse *Mbp* forward 5′ ACACACGAGAACTACCCATT ATGG 3′,

reverse 5′ AGAAATGGACTACTGGGTTTTCATCT 3′

Mouse *Tspan2* forward 5′ TGCGGTGCATCAAGTATCTG 3′,

reverse 5′ ATAACGGCTGATCCGGCTA 3′

Mouse *Cldn11* forward 5′ GTGGTGGGTTTCGTCAC GAG 3′,

reverse 5′ CGTCCATTTTTCGGCAGGTG 3′

Mouse *Mog* forward 5′ CTGTTTGTTATTGTGCCTGTT CTTG 3′,

reverse 5′ AGTCTTCGGTGCAGCCAGTT 3′

Mouse *Mag* forward 5′ GGTGTTGAGGGAGGCAGTTG 3′,

reverse 5′ CGTTCTCTGCTAGGCAAGCA 3′

Mouse *Shank3* forward 5′ TGGTTGGCAAGAGATCCAT 3′,

reverse 5′ TTGGCCCCATAGAACAAAAG 3′

Mouse *Gpr27* forward 5′ GAAGAGGCTGTGCAAGA TGTT 3′,

reverse 5′ AGCTCCCGGTTGAAGAGGA 3′

Mouse *Gpr85* forward 5′ ATGCAGCCGACAACATTT TGC 3′,

reverse 5′ CAGGTGGAGCCATTTTTGACA 3′

Mouse *Gpr173* forward 5′ CTGCACAAGGCTCCTTA CTAC 3′,

reverse 5′ CAGCCATAAAGGCCACAATCTTA 3′

Mouse *Gapdh* forward 5′ GGCATTGCTCTCAATGACAA 3′,

reverse 5′ CCCTGTTGCTGTAGCCGTAT 3′

Rat *Gpr27* forward 5′ GAAGAGGCTGTGCAAGATGTT 3′,

reverse 5′ AGCTCCCGGTTGAAGAGGA 3′

Rat *Gpr85* forward 5′ TCAGCGTCACCAGATACTTAGC 3′,

reverse 5′ CCAAACACGTCCAAAAGGTCA 3′

Rat *Gpr173* forward 5′ CTGCACAAGGCTCCTTACTAC 3′,

reverse 5′ CAGCCATAAAGGCCACAATCTTA 3′

Rat *Gapdh* forward 5′ GGATACTGAGAGCAAGAGAGA 3′,

reverse 5′ TTATGGGGTCTGGGATGGAA 3′

### cDNA Constructs

The full-length mouse *Gpr85* cDNA was PCR amplified from mouse brain cDNA library and subcloned into pRK5-Myc plasmid (WT and Δ*C* constructs). Mutagenesis reaction was performed using QuikChange II XL site-directed mutagenesis kit (Agilent Technologies) according to the manufacturer’s instructions to generate pRK5-Myc-GPR85 M152T construct (forward 5′ GGGGAATGCCGTGGCCACGGACAGAG 3′ and reverse 5′ CTCTGTCCGTGGCCACGGCATTCCCC 3′ primers). The pRK5-HA-Shank3 construct was described previously (Choi et al., 2015b). The construct contains a HA-tag followed by the full-length rat *Shank3* mRNA (NM_021676.1) with entire coding region and 3′UTR.

### Neuron Culture, Drug Treatment, Transfection, and Immunocytochemistry

Cultured cortical and hippocampal neurons were prepared from embryonic day 18 rat brains as described previously (Lee et al., 2017c). Dissociated neurons on poly-L-lysine-coated six-well plates or coverslips were placed in a neurobasal medium supplemented with B27 (Invitrogen), 0.5 mM L-glutamine, and penicillin/streptomycin (Thermo Fisher Scientific). For the drug treatment, cultured cortical neurons at days *in vitro* (DIV) 21 were treated with either picrotoxin (50 μM, Sigma-Aldrich) or tetrodotoxin (1 μM, Alomone Labs) for an indicated period and processed for RNA extraction. For immunocytochemistry, cultured hippocampal neurons at DIV 7 were transfected with Myc-GPR85 (alone or together with HA-Shank3 construct) constructs using calcium phosphate. The neurons were fixed with 4% PFA/sucrose, permeabilized with 0.2% Triton X-100, and incubated with HA (Santa Cruz, sc-7392), Myc (Santa Cruz, sc-40; Abcam, AB9106), and PSD-95 (NeuroMab, 75-028) primary and dye-conjugated secondary antibodies (Jackson ImmunoResearch). For surface staining, the neurons were incubated with Myc antibody before the permeabilization process. Images were acquired by confocal microscopy (Zeiss, LSM780) and quantified using ImageJ software in a blinded manner. Specifically, dendritic segments (length of at least 50 μm, measured by ImageJ) of the primary or secondary branches of neurons were randomly selected by an analyzer blinded to the transfected construct, and puncta along the dendritic segments were manually counted. The results were collected from three independent experiments (i.e., three independent rounds of neuron culture to image analysis; total *n* = 16, 16, 24 neurons for WT, Δ*C*, and M152T constructs were measured, respectively).

### Quantification and Statistical Analysis

Values from at least three independent experiments were used for quantification and statistical analysis. This means that we performed at least three independent technical experiments, and we used different biological samples for each technical experiment. *P*-values were calculated by two-tailed unpaired Student’s *t*-test unless otherwise specified, using GraphPad Prism 6 software. All data are presented as the mean ± SEM. ^∗^*P* < 0.05; ^∗∗^*P* < 0.01; and ^∗∗∗^*P* < 0.001.

## Results

### Identification and Comparison of DEGs From the Transcriptome Analysis of *Shank3* TG mPFC

**FIGURE 1:**
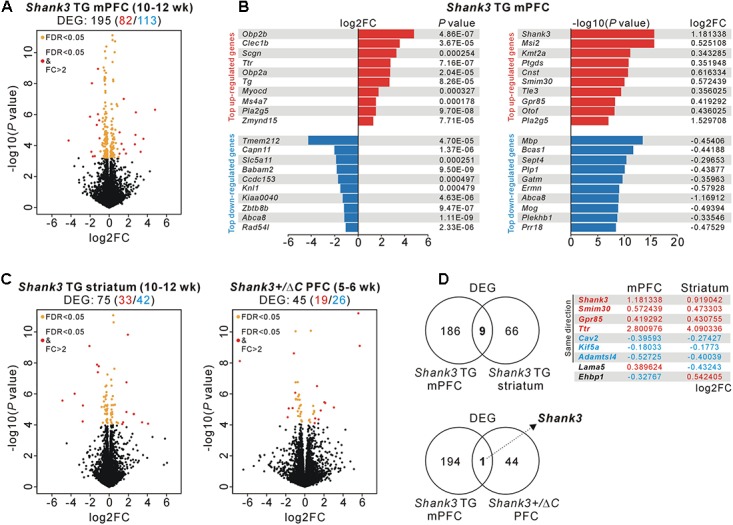
RNA-seq and DEG analyses in the *Shank3* TG mPFC and striatum and in the *Shank3^+/^*^Δ^*^C^* PFC. **(A)** Volcano plot for the mPFC RNA-seq analysis of 10- to 12-week-old *Shank3* TG mice. Differentially expressed genes (DEGs), defined by FDR < 0.05, are shown as orange (FC ≤ 2) and red (FC > 2) circles. FC, fold change. The complete lists of the RNA-seq analysis and DEGs are provided in **Supplementary Tables S2, S3**. **(B)** List of top 10 upregulated and downregulated DEGs [based on the fold changes (left) and based on the *P*-values (right)] from the mPFC RNA-seq analysis of *Shank3* TG mice. **(C)** Volcano plots for the striatum RNA-seq analysis of 10- to 12-week-old *Shank3* TG mice (left) and for the PFC RNA-seq analysis of 5- to 6-week-old *Shank3^+/^*^Δ^*^C^* mice (right). The complete lists of DEGs are provided in **Supplementary Tables S4, S5**, respectively. **(D)** The Venn diagrams show the numbers of common DEGs between the mPFC and striatum of *Shank3* TG mice (nine genes, upper) and between the mPFC of *Shank3* TG mice and the PFC of *Shank3^+/^*^Δ^*^C^* mice (only *Shank3*, lower). For the nine common DEGs between the mPFC and striatum of *Shank3* TG mice, the log_2_FC values for each brain region are shown.

To investigate molecular changes in the mPFC of *Shank3* TG mice, we performed a transcriptome analysis (RNA sequencing [RNA-seq]) of mPFC tissue from adult (10- to 12-week-old) WT and *Shank3* TG mice (**Supplementary Tables S1, S2**). We reasoned that this unbiased approach might highlight the major molecular changes or signaling pathways affected by mild Shank3 overexpression in the mPFC, as was done to reveal the altered mTORC1 signaling in the striatum of *Shank3* TG mice (Lee et al., 2017d). After applying adjusted *P* values (<0.05, Benjamini–Hochberg correction) to the transcriptome analysis, we identified 195 differentially expressed genes (DEGs) (82 upregulated and 113 downregulated) in the *Shank3* TG mPFC compared with the WT mPFC (**Figure 1A** and **Supplementary Table S3**). Based on the fold change values, odorant-binding protein 2B (*Obp2b*), C-type lectin domain family 1 member B (*Clec1b*), and secretagogin, EF-hand calcium-binding protein (*Scgn*) were the top three upregulated genes, whereas transmembrane protein 212 (*Tmem212*), calpain 11 (*Capn11*), and solute carrier family 5 member 11 (*Slc5a11*) were the top three downregulated genes (**Figure 1B**, left). In contrast, based on the *P* values, *Shank3*, Musashi RNA-binding protein 2 (*Msi2*), and lysine methyltransferase 2A (*Kmt2a*) were the top three upregulated genes, whereas myelin basic protein (*Mbp*), breast carcinoma-amplified sequence 1 (*Bcas1*), and septin 4 (*Sept4*) were the top three downregulated genes (**Figure 1B**, right).

Next, we tried to understand how specific the *Shank3* TG mPFC DEGs were by comparing them with DEGs from two recently published *Shank3*-related RNA-seq studies. One was from the striatum of adult (10- to 12-week-old) *Shank3* TG mice (Lee et al., 2017d), with which we aimed to identify both brain region-specific (i.e., mPFC- or striatum-specific) and, if any, broad (i.e., common to multiple brain regions) molecular changes in adult *Shank3* TG mice. The other was from the PFC of juvenile (5- to 6-week-old) *Shank3^+/^*^Δ^*^C^* mice (Qin et al., 2018), which are heterozygous mice for C-terminal exon 21 deletion of *Shank3*. The *Shank3^+/^*^Δ^*^C^* mice show a 50–70% reduction of Shank3 proteins in the PFC and display several autism-like behaviors, including social preference deficits and repetitive behaviors, together with molecular and functional changes of excitatory synapses in the PFC (Duffney et al., 2015). Despite the age difference (adult versus juvenile) between the mice used for the analyses, we hypothesized that we might be able to identify *Shank3* dosage-dependent molecular changes in the PFC region by comparing the RNA-seq results of *Shank3* TG mPFC and *Shank3^+/^*^Δ^*^C^* PFC.

We downloaded the raw data sets of the *Shank3* TG striatum and *Shank3^+/^*^Δ^*^C^* PFC RNA-seq analyses and re-processed them using the same protocol as for the *Shank3* TG mPFC RNA-seq analysis to compare the DEGs in greater detail. This approach identified 75 DEGs (33 upregulated and 42 downregulated) in the *Shank3* TG striatum (**Figure 1C**, left and **Supplementary Table S4**) and 45 DEGs (19 upregulated and 26 downregulated) in the *Shank3^+/^*^Δ^*^C^* PFC (**Figure 1C**, right and **Supplementary Table S5**), compared with corresponding WT controls. When we compared the DEG lists of the *Shank3* TG mPFC and striatum, nine genes were common to both, including *Shank3* (**Figure 1D**, upper). *Shank3*, small integral membrane protein 30 (*Smim30*), G protein-coupled receptor 85 (*Gpr85*), and transthyretin (*Ttr*) were upregulated, whereas caveolin 2 (*Cav2*), kinesin family member 5A (*Kif5a*), and ADAMTS like 4 (*Adamtsl4*) were downregulated, in both brain regions of *Shank3* TG mice compared with WT mice. Intriguingly, in the case of two genes, laminin subunit alpha 5 (*Lama5*) and EH domain-binding protein 1 (*Ehbp1*), the directions of changes in expression were the opposite in the mPFC and striatum of *Shank3* TG mice. *Lama5* was upregulated and downregulated in the mPFC and striatum of *Shank3* TG mice compared with WT mice, respectively. Meanwhile, *Ehbp1* was downregulated and upregulated in the *Shank3* TG mPFC and striatum, respectively. In contrast to the nine common DEGs between *Shank3* TG mPFC and striatum, there was only one common DEG, *Shank3*, between the *Shank3* TG mPFC and *Shank3^+/^*^Δ^*^C^* PFC (**Figure 1D**, lower). Overall, these results indicate that the majority of the DEGs of the adult *Shank3* TG mPFC and striatum were specific to each brain region, and that there was no common DEG, except for *Shank3* itself, between the adult *Shank3* TG mPFC and the juvenile *Shank3^+/^*^Δ^*^C^* PFC.

### Decrease of Myelin-Related mRNA Levels Specifically in the mPFC of *Shank3* TG Mice

The fact that there were not many overlaps between the DEGs of three RNA-seq analyses prompted us to investigate whether the biological pathways represented by them could be different. Even though the gene identities of DEGs were different, it is still possible that they could be involved in the same or similar biological pathways. To test this, we performed GO and KEGG pathway analyses for the DEGs of the *Shank3* TG mPFC, striatum, and *Shank3^+/^*^Δ^*^C^* PFC.

**FIGURE 2:**
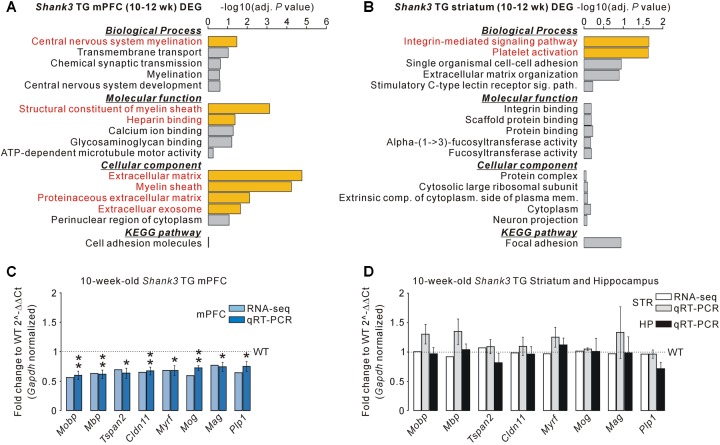
Decreased mRNA levels of myelin-related genes specifically in the mPFC, but not striatum and hippocampus, of *Shank3* TG mice. **(A)** Gene ontology (GO) and Kyoto Encyclopedia of Genes and Genomes (KEGG) pathway analyses of the DEGs of the *Shank3* TG mPFC. Significant terms (Benjamini adjusted *P*-value < 0.05) are highlighted in red. The complete list of analyses is provided in **Supplementary Table S6**. **(B)** GO and KEGG pathway analyses of the DEGs of the *Shank3* TG striatum. The complete list of analyses is provided in **Supplementary Table S7**. **(C)** qRT-PCR validation of the eight myelin-related DEGs decreased in the *Shank3* TG mPFC (dark blue bar) compared with WT mice (*n* = 5 animals per genotype; unpaired two-tailed Student’s *t*-test). The fold change value for each gene from the mPFC RNA-seq analysis is also shown (light blue bar). **(D)** qRT-PCR analysis of the eight myelin-related DEGs of the *Shank3* TG mPFC in the *Shank3* TG striatum (STR, gray bar) and hippocampus (HP, black bar) compared with WT mice (*n* = 4 animals per genotype; unpaired two-tailed Student’s *t*-test). The fold change value for each gene from the striatum RNA-seq analysis is also shown (white bar). Data are presented as mean ± SEM. ^∗^*P* < 0.05 and ^∗∗^*P* < 0.01.

For the 195 DEGs of the adult *Shank3* TG mPFC, “central nervous system myelination” in the biological process category, “structural constituent of myelin sheath” and “heparin binding” in the molecular function category, and “extracellular matrix,” “myelin sheath,” “proteinaceous extracellular matrix,” and “extracellular exosome” in the cellular component category were observed to be significant (**Figure 2A** and **Supplementary Table S6**). For the 75 DEGs of the adult *Shank3* TG striatum, “integrin-mediated signaling pathway” and “platelet activation” in the biological process category were significant, but there was no significant term in the molecular function, cellular component, or KEGG category (**Figure 2B** and **Supplementary Table S7**). For the 45 DEGs of the juvenile *Shank3^+/^*^Δ^*^C^* PFC, we did not find any significant term in the categories, possibly because the number of genes was too small (**Supplementary Table S8**). The results suggest that not only the identities of genes but also the representative biological pathways of the DEGs of the *Shank3* TG mPFC, striatum, and *Shank3^+/^*^Δ^*^C^* PFC were largely different.

Gene ontology analysis revealed that the myelin-related genes were enriched specifically in the DEGs of the *Shank3* TG mPFC. Indeed, when analyzing the list of *Shank3* TG mPFC DEGs, we identified 27 myelin-related genes (see methods for the definition of myelin-related genes), most of which (25 of 27) were downregulated in the mPFC of *Shank3* TG mice compared with WT mice (**Supplementary Table S9**). We validated eight of the decreased myelin-related DEGs (*Mobp*, *Mbp*, *Tspan2*, *Cldn11*, *Myrf*, *Mog*, *Mag*, and *Plp1*) by qRT-PCR analyses of the *Shank3* TG mPFC (**Figure 2C**). We also confirmed that the mRNA levels of eight validated myelin-related genes were not altered in the striatum and hippocampus of *Shank3* TG mice compared with WT mice (**Figure 2D**). These results indicate that the mRNA levels of myelin-related genes were altered specifically in the mPFC, but not in the striatum or the hippocampus, of adult *Shank3* TG mice. None of the 27 myelin-related DEGs of the *Shank3* TG mPFC were found in the *Shank3^+/^*^Δ^*^C^* PFC DEGs, suggesting the normal expression of the genes in *Shank3^+/^*^Δ^*^C^* mice (**Supplementary Table S5**). However, direct qRT-PCR validations are necessary to confirm this.

### Increase of *Gpr85* mRNA Levels in the Multiple Brain Regions of *Shank3* TG Mice

Next, we attempted to identify whether there were any commonly altered DEGs among the multiple brain regions of *Shank3* TG mice, which might provide additional insights into the molecular pathophysiology of *Shank3* overexpression. Therefore, we re-evaluated the nine, including *Shank3*, shared DEGs between the *Shank3* TG mPFC and striatum (**Figure 1D**, upper). Among them, *Gpr85* (also called *Sreb2* for superconserved receptor expressed in brain 2), an upregulated DEG in both mPFC and striatum of *Shank3* TG mice, was significant, because of its known associations with SCZ and ASDs (Matsumoto et al., 2008; Fujita-Jimbo et al., 2015).

**FIGURE 3:**
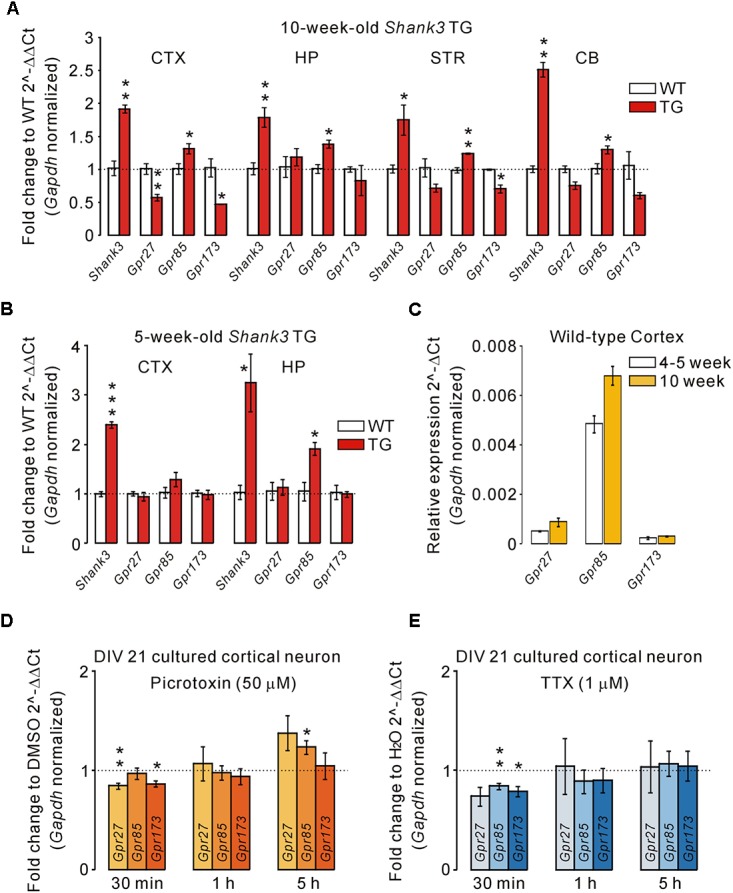
Altered mRNA levels of *Gpr27*, *Gpr85*, and *Gpr173* in multiple brain regions of *Shank3* TG mice. **(A)** qRT-PCR analysis of *Gpr27*, *Gpr85*, and *Gpr173* in the adult *Shank3* TG cortex (CTX), hippocampus (HP), striatum (STR), and cerebellum (CB) compared with WT mice (*n* = 4 animals per genotype; unpaired two-tailed Student’s *t*-test). *Shank3* was used as a positive control. **(B)** qRT-PCR analysis of *Gpr27*, *Gpr85*, and *Gpr173* in the juvenile *Shank3* TG cortex and hippocampus compared with WT mice (*n* = 4 animals per genotype; unpaired two-tailed Student’s *t*-test). **(C)** qRT-PCR analysis of the relative expression of *Gpr27*, *Gpr85*, and *Gpr173* between the cortical tissues of juvenile and adult WT mice (*n* = 3 animals). **(D)** qRT-PCR analysis of *Gpr27*, *Gpr85*, and *Gpr173* in the cultured cortical neurons of days *in vitro* (DIV) 21 treated with picrotoxin for the indicated periods of time (*n* = 5 biological replicates; unpaired two-tailed Student’s *t*-test). Dimethyl sulfoxide (DMSO) was a vehicle control. h, hour. **(E)** qRT-PCR analysis of *Gpr27*, *Gpr85*, and *Gpr173* in the neurons treated with tetrodotoxin (TTX) for the indicated periods (*n* = 4 biological replicates; unpaired two-tailed Student’s *t*-test). H_2_O was a vehicle control. Data are presented as mean ± SEM. ^∗^*P* < 0.05, ^∗∗^*P* < 0.01, and ^∗∗∗^*P* < 0.001.

There are three members in the *Sreb* gene family, *Sreb1* (*Gpr27*), *Sreb2* (*Gpr85*), and *Sreb3* (*Gpr173*), which have previously been shown to be expressed in the central nervous system (Matsumoto et al., 2000, 2005). Thus, we performed qRT-PCR for *Gpr27*, *Gpr85*, and *Gpr173* in the cortex, hippocampus, striatum, and cerebellum of adult (10-week-old) WT and *Shank3* TG mice (**Figure 3A**). We found that the mRNA levels of *Gpr85* were significantly upregulated in the four brain regions of *Shank3* TG mice compared with WT mice. Intriguingly, however, *Gpr27* and *Gpr173* mRNAs showed trends of downregulation in the four brain regions of *Shank3* TG mice. Specifically, *Gpr27* mRNA was significantly downregulated in the cortex, and *Gpr173* mRNA was significantly downregulated in the cortex and striatum of *Shank3* TG mice (**Figure 3A**). When we performed qRT-PCR analysis of the juvenile (5-week-old) WT and *Shank3* TG cortex and hippocampus, *Gpr85* mRNA was found to be significantly upregulated in the hippocampus, but not in the cortex, of *Shank3* TG mice, whereas *Gpr27* and *Gpr173* showed normal expression in both brain regions, suggesting age-dependent expression changes in *Gpr27*, *Gpr85*, and *Gpr173* in *Shank3* TG brains (**Figure 3B**). *Gpr27*, *Gpr85*, and *Gpr173* mRNA levels were slightly higher in the adult stage compared with the juvenile stage of the WT cortex (**Figure 3C**).

Next, we investigated what the potential mechanisms underlying the altered expression of *Gpr27*, *Gpr85*, and *Gpr173* in *Shank3* TG brains could be. It has been previously reported that Shank3 proteins undergo synapse-to-nucleus shuttling in an activity-dependent manner, and that Shank3 proteins may regulate the expression of several genes in the nucleus (Grabrucker et al., 2014). However, *Gpr27*, *Gpr85*, and *Gpr173* were not in the list of potential “Shank3 target genes” reported in the study. Therefore, we tested whether the neuronal activity could regulate *Gpr27*, *Gpr85*, and *Gpr173* expression. We previously showed increased excitatory, but decreased inhibitory, synaptic function, and spontaneous seizures in *Shank3* TG mice (Han et al., 2013), which suggests increased neuronal activity. We treated cultured cortical neurons of DIV 21 with either picrotoxin (a blocker for inhibitory GABA_A_ receptor) or tetrodotoxin (TTX, a sodium channel blocker inhibiting action potential firing) for three different time periods (30 min, 1 h, and 5 h) to increase and decrease neuronal activity, respectively, and measured the mRNA levels of *Gpr27*, *Gpr85*, and *Gpr173*. For the picrotoxin treatment, we found that *Gpr27* and *Gpr173* mRNAs were decreased when neurons were treated for 30 min, whereas *Gpr85* mRNA was increased when neurons were treated for 5 h (**Figure 3D**). For TTX treatment, *Gpr85* and *Gpr173* mRNAs were decreased when neurons were treated for 30 min, but there was no change in the mRNA levels in the rest of conditions (**Figure 3E**). Taken together, these results suggest that the mRNA levels of *Gpr85* and its family members, *Gpr27* and *Gpr173*, were altered in the multiple brain regions of adult *Shank3* TG mice, possibly due to increased neuronal activity.

### Negative Regulation of Excitatory Synapse Number by GPR85 in Cultured Hippocampal Neurons

GPR85 is an orphan G protein-coupled receptor (GPCR), and its molecular functions in neurons remain largely unknown. Nevertheless, a recent study showed that GPR85 could interact and co-localize with a core excitatory synaptic protein, PSD-95, in neurons (Fujita-Jimbo et al., 2015). As PSD-95 forms a protein complex with Shank3 through another protein, guanylate kinase-associated protein/synapse-associated protein-associated protein (GKAP/SAPAP) (Naisbitt et al., 1999; Kim and Sheng, 2004; Sheng and Hoogenraad, 2007; Sheng and Kim, 2011), it is possible that GPR85 can also indirectly interact with Shank3 at neuronal excitatory synapses.

**FIGURE 4:**
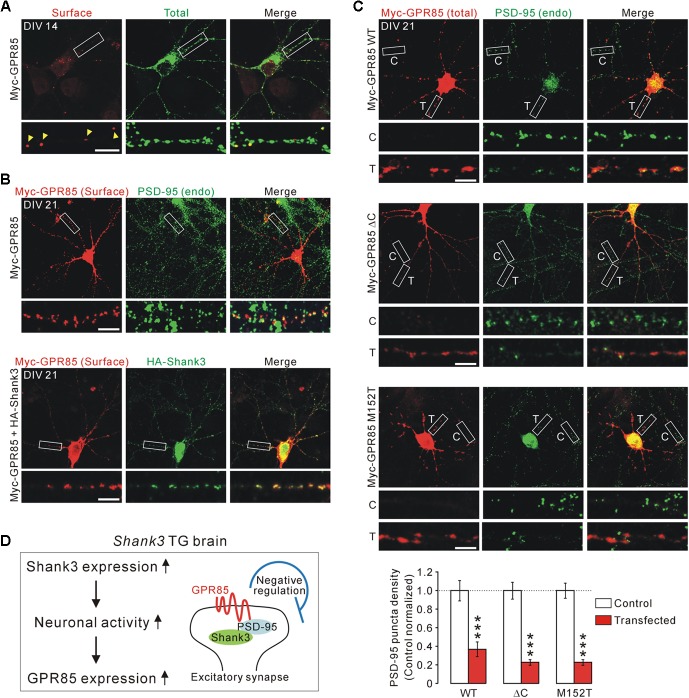
Localization and function of GPR85 in excitatory synapses of cultured hippocampal neurons. **(A)** Representative immunostaining image shows the surface and total protein expression of Myc-GPR85 in cultured hippocampal neurons of DIV 14. Scale bar, 10 μm. **(B)** Representative immunostaining images show co-localizations of surface Myc-GPR85 puncta with endogenous (endo) PSD-95 (upper) and with exogenous HA-Shank3 (lower) proteins in cultured hippocampal neurons of DIV 21. **(C)** Representative immunostaining images and quantification show the decreased density of PSD-95 in cultured hippocampal neurons of DIV 21 transfected with Myc-GPR85 constructs (WT, Δ*C*, or M152T), compared with nearby untransfected neurons (*n* = 16, 16, 24 neurons for WT, Δ*C*, and M152T constructs, respectively; unpaired two-tailed Student’s *t*-test). C, control; T, transfected. **(D)** The proposed hypothesis suggests that GPR85 expression is increased in *Shank3* TG neurons due to increased neuronal activity and that GPR85 forms a complex with Shank3 via PSD-95 to negatively regulate the number of excitatory synapses. Data are presented as mean ± SEM. ^∗∗∗^*P* < 0.001.

To test this, we generated an N-terminal Myc-tagged GPR85 construct with which surface GPR85 proteins expressed in cultured hippocampal neurons could be detected (**Figure 4A**). As previously reported (Fujita-Jimbo et al., 2015), surface GPR85 proteins were co-localized with endogenous PSD-95 proteins in cultured neurons (**Figure 4B**, upper). Moreover, when co-transfected with Shank3, the surface GPR85 and Shank3 proteins were found to be highly co-localized along neuronal dendrites, suggesting that they could form protein complexes in neurons (**Figure 4B**, lower).

Next, we investigated the functional effects of GPR85 overexpression, which might mimic its increased expression in *Shank3* TG neurons, on excitatory synapses by measuring the PSD-95 puncta density. We used three different GPR85 constructs, WT, Δ*C* without last four amino acid residues critical for PSD-95 interaction (Fujita-Jimbo et al., 2015), and M152T mutant identified in an ASD patient (Fujita-Jimbo et al., 2015). We transfected each of the GPR85 constructs to culture hippocampal neurons of DIV 7 and fixed the neurons and immunostained for PSD-95 at DIV 21. We found that the neurons transfected with GPR85 WT had significantly less PSD-95 puncta along the dendrites compared with nearby untransfected neurons (**Figure 4C**, upper). Moreover, GPR85 Δ*C* and M152T constructs also showed decreased PSD-95 puncta density, similar to the WT construct (**Figure 4C**, middle and lower). To sum up, these results suggest that GPR85 could form a protein complex with PSD-95 and Shank3 at neuronal excitatory synapses where it likely exerts a negative effect on the synaptic development and/or maintenance (**Figure 4D**).

### GSEA of the Transcriptome Analyses of the *Shank3* TG mPFC and Striatum and of the *Shank3^+/^*^Δ^*^C^* PFC

**FIGURE 5:**
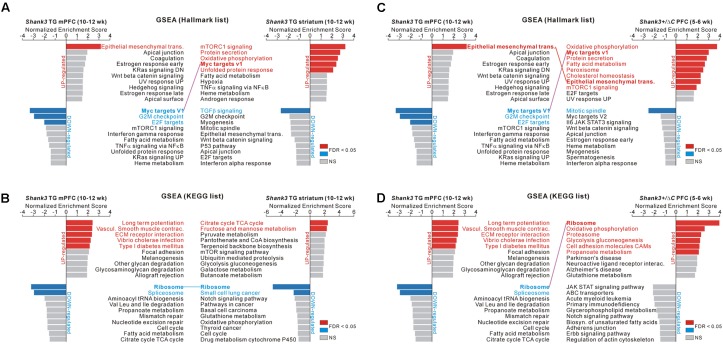
GSEA of the *Shank3* TG mPFC and striatum and *Shank3^+/^*^Δ^*^C^* PFC RNA-seq results. **(A)** The bar graphs show normalized enrichment scores (NESs) of the gene set enrichment analysis (GSEA) on the hallmark gene sets for the *Shank3* TG mPFC (left) and striatum (right) RNA-seq analyses. Significant gene sets (FDR < 0.05) are highlighted in red and blue for upregulated and downregulated genes, respectively. The common term, “Myc targets V1,” between the *Shank3* TG mPFC and striatum, is connected with a purple line. **(B)** The bar graphs show NES of GSEA on the KEGG gene sets for the *Shank3* TG mPFC (left) and striatum (right) RNA-seq analyses. The common term, “ribosome,” is connected with a blue line. **(C)** The bar graphs show NES of GSEA on the hallmark gene sets for the *Shank3* TG mPFC (left) and the *Shank3^+/^*^Δ^*^C^* PFC (right). The common terms, “epithelial mesenchymal transition” and “Myc targets V1,” are connected with red and purple lines, respectively. **(D)** The bar graphs show the NES of GSEA on the KEGG gene sets for the *Shank3* TG mPFC (left) and the *Shank3^+/^*^Δ^*^C^* PFC (right). The common term, “ribosome,” is connected with a purple line. The complete list that contains the results of the GSEA is provided in **Supplementary Tables S10–S12**.

The above-mentioned results were based on the DEGs of RNA-seq analyses, which focused on significantly altered, but a small subset of, genes. We performed GSEA of the *Shank3* TG mPFC and striatum and of the *Shank3^+/^*^Δ^*^C^* PFC RNA-seq analyses to identify meaningful “molecular signatures” based on the broader or overall expression changes in the transcriptome. We applied two different groups of gene sets (hallmark and KEGG gene sets) to the three RNA-seq analyses, which revealed significantly enriched terms from each transcriptome (**Figure 5** and **Supplementary Tables S10–S12**).

In particular, we found that several enriched terms were common among the analyses of the *Shank3* TG mPFC and striatum and the *Shank3^+/^*^Δ^*^C^* PFC. First, for the hallmark gene sets, “Myc target V1” was represented by the downregulated genes of *Shank3* TG mPFC and the upregulated genes of *Shank3* TG striatum (**Figure 5A**). Second, for the KEGG gene sets, “ribosome” was represented by the downregulated genes of both *Shank3* TG mPFC and striatum (**Figure 5B**). Third, for the hallmark gene sets, “epithelial mesenchymal transition” was enriched by the upregulated genes of both *Shank3* TG mPFC and *Shank3^+/^*^Δ^*^C^* PFC, whereas “Myc target V1” was represented by the downregulated genes of *Shank3* TG mPFC and the upregulated genes of *Shank3^+/^*^Δ^*^C^* PFC (**Figure 5C**). Finally, for the KEGG gene sets, “ribosome” was enriched by the downregulated genes of *Shank3* TG mPFC and the upregulated genes of *Shank3^+/^*^Δ^*^C^* PFC (**Figure 5D**).

**FIGURE 6:**
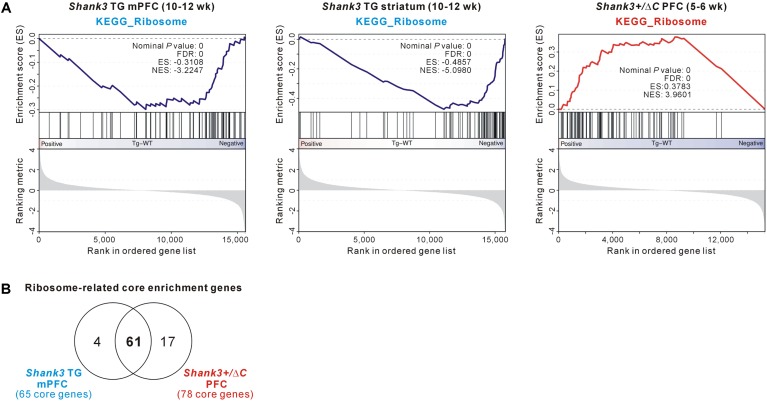
The ribosome-related molecular signature commonly detected in the *Shank3* TG mPFC and striatum and *Shank3^+/^*^Δ^*^C^* PFC RNA-seq results. **(A)** The enrichment plots of RNA-seq analyses of *Shank3* TG mPFC (left) and striatum (middle) and *Shank3^+/^*^Δ^*^C^* PFC (right) of the KEGG ribosome gene set. **(B)** The Venn diagram shows the number of common ribosome-related core enrichment genes between the *Shank3* TG mPFC (65 downregulated core genes) and the *Shank3^+/^*^Δ^*^C^* PFC (78 upregulated core genes). The lists of core genes are provided in **Supplementary Table S13**.

Among the GSEA results, we further investigated the ribosome-related gene expression changes, because those genes were downregulated in the *Shank3* TG mPFC and striatum but upregulated in the *Shank3^+/^*^Δ^*^C^* PFC (**Figure 6A**), thus possibly modulated in a *Shank3* dosage-dependent manner. Moreover, it was previously reported that neuronal mTORC1 signaling, which regulates ribosome biogenesis and function (Jastrzebski et al., 2007; Iadevaia et al., 2014), can be altered by *Shank3* overexpression or knock-down (Bidinosti et al., 2016; Lee et al., 2017d). Therefore, we compared the ribosome-related GSEA core enrichment genes of the *Shank3* TG mPFC and the *Shank3^+/^*^Δ^*^C^* PFC. There were 65 downregulated and 78 upregulated ribosome-related core genes in the *Shank3* TG mPFC and the *Shank3^+/^*^Δ^*^C^* PFC RNA-seq analyses, respectively (**Supplementary Table S13**), and surprisingly, 61 genes (74.4% of total) were found to be shared between them (**Figure 6B**). Overall, these results suggest that there could be some shared molecular signatures between the different brain regions of *Shank3* TG mice and between the PFC regions of *Shank3* TG and *Shank3^+/^*^Δ^*^C^* mice, and that ribosome-related changes could be a potential candidate.

## Discussion

In this study, we performed RNA-seq analysis of the mPFC of *Shank3* TG mice and compared the result, side by side, with the previously reported RNA-seq analyses of the striatum of *Shank3* TG mice and the PFC of *Shank3^+/^*^Δ^*^C^* mice. We found that the expression of myelin-related genes specifically decreased in the mPFC and the expression of *Gpr85* increased in multiple brain regions of *Shank3* TG mice by comparing the DEGs of different RNA-seq analyses. Moreover, the GSEA results suggested that the expression of a group of ribosome-related genes could be altered in a *Shank3* dosage-dependent manner, at least in the PFC regions of mice. One obvious limitation of our analysis is that we compared DEGs from the mPFC of “adult” *Shank3* TG mice and from the PFC of “juvenile” *Shank3^+/^*^Δ^*^C^* mice. Indeed, when we compared the transcriptomes of “adult” WT mPFC and “juvenile” WT PFC (**Supplementary Table S14**), or “adult” TG mPFC and “juvenile” WT PFC (**Supplementary Table S15**), there were about 5,300 DEGs in both cases, suggesting that developmental stages largely affect gene expression profiles (note that we therefore compared *Shank3*-dependent DEGs normalized to the age-matched WT controls). Furthermore, considering differential *Shank3* expression in brain across developmental stages (Wang et al., 2014), it is possible that DEGs of the PFC of juvenile *Shank3^+/^*^Δ^*^C^* mice can be different from those of adult *Shank3^+/^*^Δ^*^C^* mice. Therefore, further validation will be necessary to confirm that a group of ribosome-related genes of PFC are altered in a *Shank3* dosage-dependent manner. In contrast, it is also possible that additional common DEGs, other than ribosome-related genes, may be identified if we compare *Shank3* TG mice and *Shank3^+/^*^Δ^*^C^* mice of similar developmental stages.

The brain region-specific Shank3 isoform expression (Wang et al., 2014) and Shank3 interactome (Lee et al., 2017b) have been suggested to contribute to the phenotypic complexity and heterogeneity of *SHANK3*-associated brain disorders. It is also possible that such protein-level diversity of Shank3 might contribute to the brain region-specific changes of transcriptome in *Shank3* mutant mice. For example, Shank3 proteins can undergo synapse-to-nucleus shuttling in an activity-dependent manner to regulate the expression of several genes in the nucleus (Grabrucker et al., 2014). Certain Shank3 isoforms may be more preferentially targeted to the nucleus or some Shank3 interactors may be involved in the synapse-to-nucleus shuttling of Shank3. If so, each brain region could have different levels of Shank3 proteins in the nucleus. Further investigations about Shank3-dependent gene expression and its regulation will be necessary to test this intriguing hypothesis.

It was unexpected that the mRNA levels of myelin-related genes would be altered in the mPFC of adult *Shank3* TG mice. As the majority of Shank3 proteins in the brain function at the neuronal excitatory synapses, it is likely that the altered expression of myelin-related genes could be an indirect effect of Shank3 overexpression in the neurons. Indeed, it has been reported that the neuronal activity can promote oligodendrogenesis and myelination (Gibson et al., 2014; Mitew et al., 2018). However, this might not be the case for *Shank3* TG mice where the neuronal activity is expected to increase (Han et al., 2013), but the expression of myelin-related genes decreases. As an alternative possibility, various types of behavioral stress, including neonatal maternal separation, chronic social isolation, and chronic social defeat, can impair myelination in rodents, mainly in the PFC regions (Liu et al., 2012; Lehmann et al., 2017; Yang et al., 2017). Moreover, increased serum levels of stress hormone corticosterone and altered hypothalamic-pituitary-adrenal (HPA) axis have been observed in human patients and animal models of mania (Daban et al., 2005; Leussis et al., 2013). Therefore, as an animal model of mania (Han et al., 2013; Lee et al., 2018), *Shank3* TG mice might also have increased levels of stress hormones, which could secondarily affect the expression of myelin-related genes in the mPFC. Further validation of myelin-related phenotypes of the mPFC, together with measurements of stress hormone levels, in *Shank3* TG mice could be an interesting topic of the future study.

Among the common DEGs between the mPFC and striatum of adult *Shank3* TG mice, we further characterized *Gpr85* and its family members *Gpr27* and *Gpr173*. In the cortex, hippocampus, striatum, and cerebellum of adult *Shank3* TG mice, the mRNA levels of *Gpr85* increased, whereas those of *Gpr27* and *Gpr173* decreased, compared with WT mice, suggesting some compensatory changes in expression among the family members. In particular, when we treated cultured cortical neurons with picrotoxin to increase the neuronal activity and thereby possibly mimick the neurons of *Shank3* TG mice, we observed an increase in *Gpr85*, but a decrease in *Gpr27* and *Gpr173*, mRNA levels. Therefore, elevated neuronal activity could, at least in part, contribute to the altered expression of *Gpr85*, *Gpr27*, and *Gpr173* in *Shank3* TG brains. It was unexpected that, in the case of *Gpr173*, mRNA levels were also reduced under decreased neuronal activity by TTX treatment. Detailed transcriptional (or post-transcriptional) mechanisms underlying activity-dependent expression changes of *Gpr85*, *Gpr27*, and *Gpr173* need to be further investigated.

Beyond their relationship at the level of transcriptional regulation, we further investigated the functional interaction between Shank3 and GPR85 at neuronal excitatory synapses, based on the recently reported protein–protein interactions between PSD-95 and GPR85 (Fujita-Jimbo et al., 2015). Indeed, GPR85 proteins were found to be co-localized with PSD-95 and Shank3 along the neuronal dendrites, suggesting that increased GPR85 in *Shank3* TG neurons could be targeted to the excitatory synapses. To replicate this condition, we overexpressed GPR85 in cultured hippocampal neurons and found that GPR85 significantly decreased the number of excitatory synapses. Even GPR85 proteins without the C-terminal four residues or those harboring an ASD-associated variant (M152T) were nonetheless found to decrease excitatory synapses in cultured neurons, suggesting that these mutant proteins may still exert their negative effect at excitatory synapses. It is not uncommon that PSD-95-interacting proteins negatively regulate excitatory synaptic development and function. For example, Neph2/Kirrel3, an adhesion molecule interacting with PSD-95, negatively regulates excitatory synaptic transmission in the dentate granule neurons of the hippocampus (Choi et al., 2015a; Roh et al., 2017). Although the detailed underlying mechanisms need to be further investigated, we speculate that an activity-dependent increase of *Gpr85* expression, together with its negative regulation of excitatory synapses, could represent a homeostatic response in *Shank3* TG neurons. In the original paper describing the *Shank3* TG mice (Han et al., 2013), we found increased excitatory synapse number in TG neurons compared with WT neurons. We speculate that Shank3 overexpression in TG neurons increases excitatory synapse number, which in turn elevates neuronal activity to induce *Gpr85* mRNA expression. GPR85 proteins in *Shank3* TG neurons may have negative effects on excitatory synapse number, but its expression level may not be enough to fully normalize synapse number to the WT level. Meanwhile, in our GPR85 overexpression experiment, we could observe decreased synapse number because we overexpressed GPR85 very high level for a long period (2 weeks). Considering the associations of *GPR85*/*SREB2* with ASDs and SCZ (Matsumoto et al., 2008; Chen et al., 2012; Radulescu et al., 2013; Fujita-Jimbo et al., 2015), more investigations about the functional relationship between Shank3 and GPR85 would provide information on the detailed mechanisms of activity-dependent synaptic regulation and their potential implications in multiple brain disorders.

To identify meaningful molecular signatures based on broader expression changes, instead of the DEGs, we performed a GSEA of the RNA-seq results, which revealed some commonly enriched terms among the *Shank3* TG mPFC and striatum and the *Shank3^+/^*^Δ^*^C^* PFC. Notably, ribosome-related genes were downregulated in the *Shank3* TG mPFC and striatum but upregulated in the *Shank3^+/^*^Δ^*^C^* PFC, suggesting that those genes could be altered in a *Shank3* dosage-dependent manner. Indeed, when we compared the ribosome-related core enrichment genes of the *Shank3* TG mPFC and the *Shank3^+/^*^Δ^*^C^* PFC, most were shared and showed the opposite directional changes in expression in the two mice. Although it is not currently easy to explain how the level of Shank3 proteins affects the expression of a group of ribosome-related genes, changes in mTORC1 signaling, which regulates ribosome biogenesis and function (Jastrzebski et al., 2007; Iadevaia et al., 2014), might be involved. It was found that the reduced Shank3 expression decreased mTORC1 activity by increasing steady-state levels of Cdc2-like kinase 2 (CLK2) (Bidinosti et al., 2016). The CLK2 phosphorylates and activates the regulatory subunit of protein phosphatase 2A (PP2A), which in turn inhibits AKT, a positive upstream regulator of mTORC1. Moreover, we recently showed that the mTORC1 activity is decreased in the striatum of *Shank3* TG mice, possibly due to the aberrant protein interactions between Shank3 and some upstream regulators of mTORC1 (Lee et al., 2017d). A comprehensive analysis of mTORC1 activity in various brain regions of *Shank3* mutant mice, together with the direct measurement of ribosomal quantity (both mRNA and protein) and activity, could provide important clues regarding the detailed mechanisms behind this. In this regard, it is worth noting that in a recent quantitative proteomic analysis of the striatal PSD of *Shank3* mutant mice (*Shank3*Δ*11^−/−^*), the levels of several ribosomal proteins were increased compared with WT mice (Reim et al., 2017). Shank3 proteins are coupled with metabotropic glutamate receptors (mGluRs) via Homer (Tu et al., 1999) and regulate their synaptic expression and signaling (Verpelli et al., 2011; Vicidomini et al., 2016; Wang et al., 2016). mGluR signaling is a key regulator of synaptic local translation (Waung and Huber, 2009; Luscher and Huber, 2010). Therefore, abnormal mGluR signaling and altered levels of ribosomal proteins may together lead to changes in synaptic mRNA translation in *Shank3* mutant mice, which can be an important topic of the future study.

## Conclusion

In conclusion, using unbiased transcriptome analyses, our study reveals both brain region-specific and broad, previously unidentified molecular changes in *Shank3*-overexpressing mice. These results further elucidate the complexity and heterogeneity of the molecular pathophysiology of *SHANK3*-associated brain disorders and also highlight the need for subsequent studies on relevant molecular pathways, which could be applied, in the long term, for the development of the better therapeutic approaches.

## Author Contributions

CJ, JR, SK, YZ, YL, YK, and KH designed and performed the experiments. HK and KH analyzed and interpreted the data, and wrote the paper. All authors read and approved the manuscript.

## Conflict of Interest Statement

The authors declare that the research was conducted in the absence of any commercial or financial relationships that could be construed as a potential conflict of interest.

## Footnotes

1 http://www.informatics.jax.org/homology.shtml

2 http://amigo.geneontology.org/amigo

3 http://software.broadinstitute.org/gsea

4 http://software.broadinstitute.org/gsea/msigdb
